# Prognostic nomogram of xerostomia for patients with nasopharyngeal carcinoma after intensity-modulated radiotherapy

**DOI:** 10.18632/aging.102717

**Published:** 2020-01-31

**Authors:** Xin-Bin Pan, Yang Liu, Ling Li, Song Qu, Long Chen, Shi-Xiong Liang, Kai-Hua Chen, Zhong-Guo Liang, Xiao-Dong Zhu

**Affiliations:** 1 Department of Radiation Oncology, Guangxi Medical University Cancer Hospital, Nanning, Guangxi 530021, P.R. China; * Equal contribution

**Keywords:** nasopharyngeal carcinoma, intensity-modulated radiotherapy, xerostomia

## Abstract

Xerostomia is a common radiation-induced late complication after radiotherapy. Identifying predictive factors for xerostomia will lead to better treatments and improve the quality of life. This study was conducted to establish an effective predictive nomogram for xerostomia by assessing stage I-IVb (AJCC 7th edition) NPC patients between September 2015 and March 2016. Xerostomia was evaluated via the RTOG/EORTC system. The primary endpoint was grade 2-3 xerostomia 1 year after treatment. The predictive factors for xerostomia were analysed using logistic regression analysis. A nomogram was constructed based on combining the predictors and clinical variables. In total, 102 patients with grade 0-1 xerostomia and 93 patients with grade 2-3 xerostomia were included. The independent predictive factors for xerostomia were V25, V30, V35, and V45 of the ipsilateral parotid gland and mean dose of the contralateral parotid gland. The calibration plot for the probability of xerostomia showed good agreement between prediction by the nomogram and actual observation. The concordance index of the nomogram for predicting xerostomia was 0.796 (95% CI: 0.735-0.857, P <0.001), which was higher than any single dosimetric parameter. Our results indicated that the nomogram provided a more accurate prediction of grade 2-3 xerostomia 1 year after treatment.

## INTRODUCTION

Nasopharyngeal carcinoma (NPC) is an endemic cancer in Southern China [1, 2]. NPC is a radiosensitive malignant tumor. Radiotherapy is the primary treatment modality for NPC. Xerostomia is a common radiation-induced late complication after radiotherapy [3]. The incidence of clinically significant xerostomia was more than 30% after intensity-modulated radiotherapy (IMRT) [4–6]. Xerostomia degrades the quality of life by disrupting eating, sleep, speech, and communication [7, 8]. Therefore, identifying predictive factors for xerostomia will lead to better treatments for patients with risks of severe xerostomia and improve the quality of life [9–11].

Currently, the potential predictive factors of xerostomia after IMRT in NPC patients remain unclear. Previous studies reported that the mean dose of the parotid glands was a predictor of xerostomia in patients with head and neck squamous cell carcinomas [12–17]; however, the most appropriate cut-off points for the mean dose differed significantly in previous studies [18–21]. Moreover, the xerostomia clinical factors require further assessment, including age, sex, pathology, tumour stage, chemotherapy, and volume of the parotid glands. Therefore, a distinct predictive model based on the dosimetric parameters and clinical variables would provide more accurate predictions than single parameters.

This study was conducted to identify the potential predictive dosimetric parameters of xerostomia and establish a predictive nomogram in NPC patients receiving IMRT.

## RESULTS

### Patient characteristics

**Figure 1:**
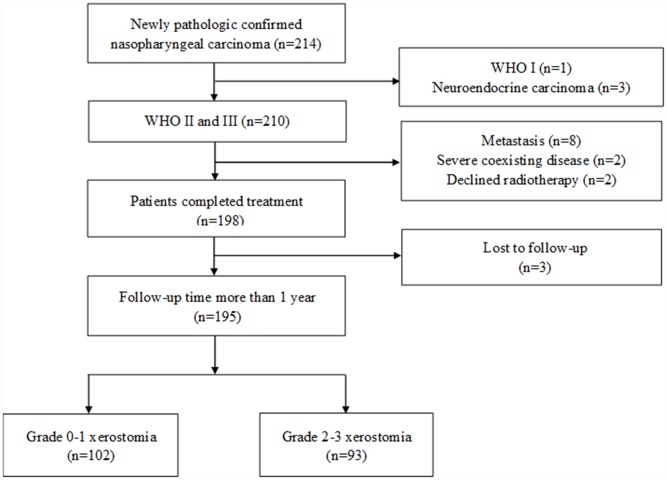
**Flowchart depicting patient selection.**

This study included 195 patients: 102 patients in the grade 0-1 xerostomia group and 93 patients in the grade 2-3 xerostomia group. A flowchart is shown in Figure 1. The patient characteristics are shown in Table 1. The baseline clinical characteristics are balanced between the two group, except the N stage and the AJCC stage. All the patients received follow up for >12 months.

**Table 1:** Patient characteristics for grade 0-1 and grade 2-3 xerostomia 1 year post-treatment.

	**Grade 0-1 xerostomia (n=102)**	**Grade 2-3 xerostomia (n=93)**	P
Age at diagnosis (years)			0.152
Median	47	47	
Range	15-62	24-74	
Sex			0.144
Male	80(78.4%)	64(68.8%)	
Female	22(21.6%)	29(31.2%)	
T stage			0.147
T1	9(8.8%)	3(3.2%)	
T2	33(32.4%)	29(31.2%)	
T3	26(25.5%)	18(19.4%)	
T4	34(33.3%)	43(46.2%)	
N stage			0.010
N0	10(9.8%)	1(1.1%)	
N1	47(46.1%)	33(35.5%)	
N2	35(34.3%)	44(47.3%)	
N3	10(9.8%)	15(16.1%)	
AJCC stage			0.007
I	4(3.9%)	0(0.0%)	
II	27(26.5%)	11(11.8%)	
III	31(30.4%)	29(31.2%)	
IVa-b	40(39.2%)	53(57.0%)	
Pathology			0.383
WHO II	15(14.7%)	9(9.7%)	
WHO III	87(85.3%)	84(90.3%)	
BMI (kg/m^2^)			0.585
<18.5	5(4.9%)	5(5.4%)	
18.5-22.9	53(52.0%)	41(44.1%)	
22.9-27.5	40(39.2%)	40(43.0%)	
≥27.5	4(3.9%)	7(7.5%)	
Chemotherapy			0.248
No	13(12.7%)	7(7.5%)	
Yes	89(87.3%)	86(92.5%)	

WHO: World Health Organization. AJCC: the American Joint Committee on Cancer.

BMI: body mass index.

### Predictors for xerostomia

The dosimetry parameters were comparable for the patients in the grade 0-1 xerostomia group and the grade 2-3 xerostomia group (Table 2). The predicted probability for the dosimetry parameters is listed in Table 2. The results indicate that each single parameter has a low assessment ability, which is less than 0.700.

**Table 2:** Predicted probability of the dosimetry parameters for grade 2-3 xerostomia 1 year post-treatment.

	**Dosimetry parameters (median, range)**			**Predicted probability**		
	**Grade 0-1 xerostomia**	**Grade 2-3 xerostomia**	**P**	**AUC**	**95% CI**	**P**
Contralateral parotid gland						
CPG.Dmean (Gy)	36.92 (29.75-48.57)	38.43 (29.32-74.10)	0.522	0.671	0.595-0.747	<0.001
CPG.V10 (%)	100.00 (97.65-100.00)	100.00 (99.72-100.00)	0.473	0.571	0.504-0.638	0.020
CPG.V15 (%)	97.80 (84.38-100.00)	99.15 (90.72-100.00)	0.403	0.630	0.552-0.708	0.001
CPG.V20 (%)	84.86 (63.48-99.62)	88.81 (73.48-100.00)	0.328	0.664	0.588-0.740	<0.001
CPG.V25 (%)	67.04 (49.65-89.87)	69.93 (54.55-100.00)	0.347	0.658	0.581-0.734	<0.001
CPG.V30 (%)	54.96 (35.61-74.65)	58.16 (39.52-100.00)	0.366	0.661	0.584-0.737	<0.001
CPG.V35 (%)	46.94 (26.11-68.25)	49.54 (30.20-100.00)	0.386	0.664	0.587-0.742	<0.001
CPG.V40 (%)	40.51 (19.38-62.27)	42.24 (21.73-100.00)	0.426	0.640	0.561-0.718	<0.001
CPG.V45 (%)	34.20 (14.87-56.32)	36.50 (14.44-100.00)	0.446	0.633	0.555-0.712	0.001
CPG.V50 (%)	27.66 (11.62-50.04)	30.07 (7.77-100.00)	0.446	0.629	0.551-0.708	0.001
CPG.V55 (%)	20.50 (6.54-43.24)	23.49 (2.59-99.82)	0.446	0.638	0.560-0.715	<0.001
CPG.Volume (cm^3^)	30.02 (14.05-52.77)	25.97 (12.85-47.84)	0.378	0.612	0.532-0.692	0.003
Ipsilateral parotid gland:						
IPG.Dmean (Gy)	35.51 (20.80-41.05)	36.56(27.47-44.31)	0.382	0.646	0.569-0.723	<0.001
IPG.V10 (%)	100.00 (95.77-100.00)	100.00(99.11-100.00)	0.477	0.502	0.430-0.574	0.478
IPG.V15 (%)	97.10 (84.55-100.00)	98.25(90.06-100.00)	0.482	0.604	0.524-0.683	0.006
IPG.V20 (%)	82.14 (50.72-95.72)	86.25(71.63-99.15)	0.426	0.666	0.591-0.742	<0.001
IPG.V25 (%)	63.60 (19.29-77.66)	67.99(48.79-90.21)	0.406	0.689	0.616-0.763	<0.001
IPG.V30 (%)	52.13 (6.91-67.05)	54.59(32.02-76.90)	0.446	0.687	0.613-0.761	<0.001
IPG.V35 (%)	43.94 (2.92-54.41)	46.71(22.27-64.84)	0.446	0.675	0.598-0.751	<0.001
IPG.V40 (%)	37.54 (1.40-47.62)	39.81(15.21-54.48)	0.466	0.647	0.569-0.726	<0.001
IPG.V45 (%)	31.11 (0.61-43.21)	33.09(9.58-47.35)	0.426	0.607	0.527-0.686	0.005
IPG.V50 (%)	24.08 (0.22-38.66)	25.56(5.14-40.81)	0.426	0.589	0.508-0.669	0.016
IPG.V55 (%)	16.30 (0.08-33.58)	18.25(2.59-33.27)	0.446	0.580	0.499-0.660	0.028
IPG.Volume (cm^3^)	29.47 (14.63-58.54)	24.34(15.02-47.39)	0.480	0.597	0.516-0.677	0.010

CPG: contralateral parotid gland. IPG: ipsilateral parotid gland. Dmean: mean dose. AUC: area under curve. CI: confidence interval.

**Figure 2:**
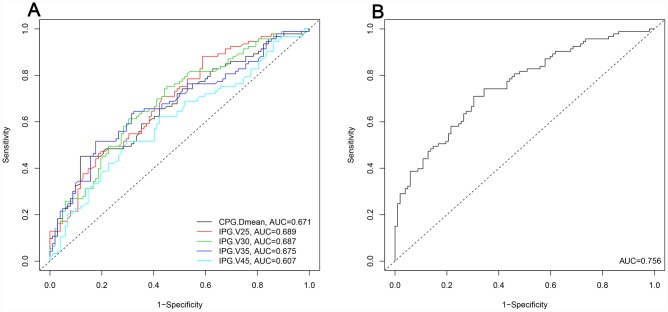
**Predicted probability of the independent dosimetry parameters and combined predictors for grade 2-3 xerostomia at the 1 year follow-up.** (**A**): Predicted probability of the independent dosimetry parameters. (**B**): Predicted probability of the combined predictors. CPG: contralateral parotid gland. IPG: ipsilateral parotid gland. Dmean: mean dose. AUC: area under the curve.

Multivariate analysis of the logistic regression analysis revealed that V25, V30, V35, and V45 of the ipsilateral parotid gland and mean dose to the contralateral parotid gland were independent predictive factors for grade 2-3 xerostomia 1 year after treatment (Table 3). The predicted probability for these independent parameters is shown in Figure 2A. The cut-off points of V25, V30, V35, and V45 of the ipsilateral parotid gland and the contralateral parotid gland mean dose are 62.23%, 53.59%, 46.62%, 33.02%, and 39.63 Gy, respectively. The AUC of the combined predictor (mx) for grade 2-3 xerostomia 1 year after treatment is 0.756 (95% CI: 0.689-0.823, P <0.001) (Figure 2B).

**Table 3:** Logistic regression for grade 2-3 xerostomia 1 year post-treatment.

	**Univariate**			**Multivariate**		
	**OR**	**95% CI**	**P**	**OR**	**95% CI**	**P**
CPG.Dmean	2.82	1.59-4.99	<0.001	2.04	1.16-3.61	0.014
IPG.V25	2.39	1.61-3.55	<0.001	6.95	1.83-26.42	0.004
IPG.V30	2.42	1.55-3.79	<0.001	0.02	0.01-0.36	0.008
IPG.V35	2.32	1.46-3.68	<0.001	77.44	5.06-1184.51	0.002
IPG.V45	1.45	1.05-2.00	0.023	0.19	0.0-0.56	0.003

CPG: contralateral parotid gland. IPG: ipsilateral parotid gland. Dmean: mean dose. OR: odds ratio. CI: confidence interval.

### Prognostic nomogram for xerostomia

**Figure 3:**
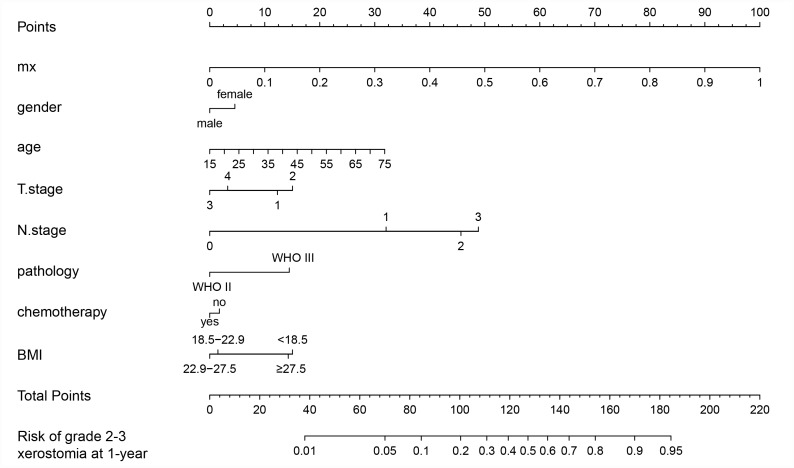
**Nomogram of grade 2-3 xerostomia at the 1 year follow-up.**

**Figure 4:**
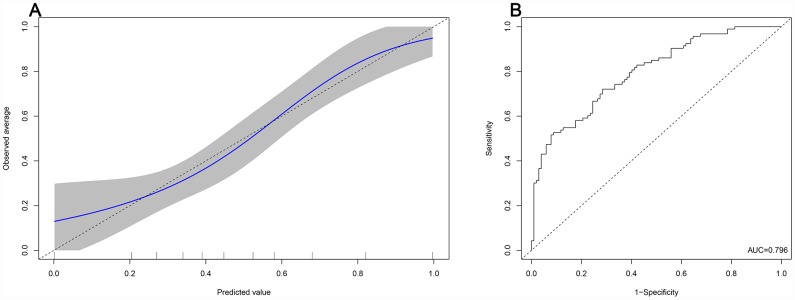
**Predicted probability of the nomogram.** (**A**): The calibration plot of the nomogram for predicting grade 2-3 xerostomia at the 1 year follow-up. (**B**): Area under the curve of the nomogram.

The prognostic nomogram that integrated all the clinical variables and combined predictors is shown in Figure 3. The concordance index of the prognostic nomogram was 0.796 (95% CI: 0.735-0.857, P <0.001). The calibration plot for the probability of grade 2-3 xerostomia 1 year after treatment showed an optimal agreement between the predictive value from the nomogram and actual observation (Figure 4A). The predicted probability of the prognostic nomogram is 0.796 (95% CI: 0.734-0.857, P <0.001) (Figure 4B).

## DISCUSSION

The dosimetry parameters of the parotid glands have been commonly used for the prediction of xerostomia. Current controversies on the different dosimetry parameters focus on which dosimetric parameter is an independent predictive factor of xerostomia and whether additional risk factors, other than the dosimetric parameters, are important. These factors are not specifically developed for NPC patients, but rather for patients with head and neck squamous cell carcinomas. The predictive accuracy of these factors might be affected by these questions.

This study found that the predictive ability of each single dosimetric parameter ranged from 0.502 to 0.689 in AUC, which indicates low discrimination. Similarly, a prospective cohort analysis revealed the same results [22]; however, in the previous study, only V60 of the contralateral parotid gland (95% CI: 0.99-1.07, P = 0.080) was associated with near statistical significance with the presence of xerostomia. In contrast, the predictive probability of the combined predictors based on the results of multivariate analysis of the logistic regression analysis significantly increased to 0.756 in this study. Moreover, the predictive nomogram constructed based on the combined predictors and clinical variables performed well in predicting grade 2-3 xerostomia (AUC = 0.796), and the prediction was supported by the C-index and the calibration curve. Compared to the prospective study, the current retrospective study provides a better predictive model with high accuracy for xerostomia.

Several studies reported a correlation between the mean dose of the parotid glands and xerostomia in patients with head and neck squamous cell carcinomas 12–17]. Our study also revealed a similar result: a mean dose ≥39.63 Gy to the contralateral parotid gland was a risk factor for grade 2-3 xerostomia; however, Sommat et al [22] found that the mean dose to the parotid glands was not a predictive factor. Possible reasons for these opposing results may be that all patients in the study of Sommat et al [22] had locoregionally advanced NPC and the dose distribution of the glands did not differ among the patients. In contrast, our cohort included early stage patients who received low dose treatment.

A mean dose <26 Gy for at least one parotid gland is recommended as a planning goal according to previous studies [20, 21]. Pre-treatment salivary flow rates can be completely recovered using this dose; however, this dose is hard to achieve in NPC patients. Because the tumour is close to the parotid glands, an overzealous effort in reducing doses to the parotid glands might decrease planning doses in the target volume, which is a risk for disease recurrence. Thus, the mean dose to the parotid glands in NPC patients was consistently more than 30 Gy. Our study indicated that the cut-off value of the mean dose to the contralateral parotid gland was 39.63 Gy. Similarly, Sommat et al [22] reported that the average mean dose to the parotid glands was in excess of 41 Gy. Other studies revealed a dose range from 31.3 to 38 Gy [23–25].

In clinical practice, a V30 of <50% of the parotid gland is a commonly used criterion instead of the mean dose to the parotid glands. In this study, a V30 <53.59% to the ipsilateral parotid gland was a protective factor for xerostomia. Our results demonstrated that a V30 <50% to the parotid gland was a reasonable criterion; however, the addition of V25 <62.23% and V35 <46.62% criteria to the planning evaluation may further improve parotid function preservation. As indicated by the results of the multivariate logistic regression analysis, the predicted probability of V25 was the most accurate (AUC = 0.689).

Xerostomia was commonly observed 2 months after radiotherapy, and continuously improved thereafter [26]. Approximately 60% of patients recovered at least 25% of their baseline saliva secretion 1 year post treatment [27] and their salivary function became stable after 1 year. The incidence of xerostomia at the 1 year follow-up was similar to the 2 year [22]. Many patients may not recover salivary flow, and xerostomia remains consistent over time [28]. Therefore, xerostomia assessment 1 year after IMRT was reasonable in this study.

This study revealed that the incidence of grade 2-3 xerostomia at the 1 year follow-up was 47.69%, which indicates that xerostomia remained a significant long term complication after IMRT; however, the incidence of xerostomia varied among previous studies. Our results are consistent with other reports. McDowell et al [6] reported that 46.7% of patients had grade 2-3 xerostomia after IMRT at the 4 year follow-up. Another study found that the incidence of grade 2-3 xerostomia was 43% [5]. In contrast, other studies reported that the incidence of grade 2-3 xerostomia ranged from 20.1% to 33% [4, 23, 29]. Possible reasons for these inconsistent findings could be differing patient inclusion criteria and follow-up time.

This study found that chemotherapy was not associated with grade 2-3 xerostomia, similarly to several other studies. Zeng et al [23] reported that chemotherapy had no impact on xerostomia in NPC patients treated with IMRT (P = 0.211). Moreover, Miah et al [5] assessed the incidence of xerostomia ≥ grade 2 between IMRT alone and concurrent chemoradiotherapy groups in 2 prospective studies. The authors found that the addition of chemotherapy to IMRT was not associated with the incidence of acute (60.3% vs 64.7%, P = 0.83) or late (34% vs 43%, P = 0.15) xerostomia. Our previous study also found no significant difference in dry mouth (P = 0.975) and sticky saliva (P = 0.358) between the radiotherapy and chemoradiotherapy groups based on patients’ self-reported xerostomia [30].

This study had several limitations. First, the nomogram was established based on data obtained from a single institution. The nomogram requires validation in another cohort. Second, this study did not assess the dosimetric parameters of the submandibular glands for xerostomia. Third, patients’ self-reported xerostomia may be a more reasonable assessment as it provides the patients’ perspective of xerostomia on quality of life, which might not be captured by physicians [31]. This study assessed xerostomia according to the RTOG/EORTC system. The subjective assessment of the RTOG/EORTC system may underestimate the severity of xerostomia compared with the patient self-reported scores [22, 32]. Therefore, the nomogram should be verified in a prospective cohort study with a patient self-reported and validated xerostomia questionnaire.

In conclusion, this study constructed a nomogram to accurately predict grade 2-3 xerostomia in NPC patients 1 year post-treatment with IMRT. Further studies are needed to verify whether the nomogram can be applied in clinical practice.

## MATERIALS AND METHODS

### Patients

This retrospective cohort study was conducted at Guangxi Medical University Cancer Hospital. NPC patients who were treated between September 2015 and March 2016 were assessed. The inclusion criteria included the following: (1) newly confirmed World Health Organization type II or III histology; (2) stage I-IVb NPC [7^th^ edition of the American Joint Committee on Cancer (AJCC)]; and (3) patients received IMRT. Exclusion criteria were as follows: (1) palliative treatment; (2) previous malignancy; (3) pregnancy or lactation; (4) previous radiotherapy, chemotherapy, or surgery (except diagnostic) to the primary tumour or lymph nodes; (5) severe coexisting diseases included heart failure, uncontrolled diabetes, severe hepatitis, and renal dysfunction; and (6) diseases that affected the secretion of salivary glands.

This study was approved by Guangxi Medical University Cancer Hospital Ethics Committee. Informed consent was obtained from all the patients. This study did not register online due to the retrospective nature.

### Treatment

IMRT was based on the International Commission on Radiation Units and Measurements Report 62 guidelines. The gross tumour volume of the nasopharynx (GTVnx) and gross tumour volume of the cervical lymph nodes (GTVnd) were quantified by using computed tomography (CT) or magnetic resonance imaging (MRI) scans. The high-risk clinical target volume (CTV1) included the GTVnx plus a 5–10 mm margin to encompass the high-risk sites of microscopic extension and the whole nasopharynx. The low-risk clinical target volume (CTV2) was defined as the CTV1 plus a 5–10 mm margin to encompass the low-risk sites of microscopic extension, including the skull base, the clivus, the sphenoid sinus, the parapharyngeal space, the pterygoid fossae, the posterior parts of the nasal cavity, the pterygopalatine fossae, the retropharyngeal nodal regions, and the elective neck area from level IB to V. The planning target volume (PTV) was defined by adding a 3 mm margin to the GTV or CTV. The prescribed radiation doses were 70.06-72.32 Gy for the PGTVnx, 66.00-72.32 Gy for the PGTVnd, 60.00-62.00 Gy for the PCTV1, and 54.00-55.80 Gy for the PCTV2.

Concurrent chemotherapy was 100 mg/m^2^ of cisplatin for 1 or 3 days with 1 cycle on days 1, 22, and 43 during radiotherapy. Induction chemotherapy included 60 mg/m² of docetaxel for 1 day, 60 mg/m^2^ of cisplatin for 1 day, and 600 mg/m^2^/day of 5-fluorouracil as a continuous intravenous infusion for 120 hours for 3 cycles.

### Dosimetric parameters

All the parotid glands were contoured based on the fusion images from the MRI-CT-Sim to reduce observer variability. No margin was added during treatment planning for the parotid glands. The dosimetric parameters were calculated from the dose-volume histograms in the radiotherapy planning system of Pinnacle³ 9.8 (Philips Co., Eindhoven, Netherlands). The pre-treatment parameters included the mean dose to the ipsilateral and the contralateral parotid glands, the volume of the ipsilateral and the contralateral parotid glands, and V10, V15, V20, V25, V30, V35, V40, V45, V50, V55 of the ipsilateral and the contralateral parotid glands.

### Xerostomia assessment

Xerostomia was assessed by physicians at 3 months, 6 months, and 12 months after treatment according to the Radiation Therapy Oncology Group/European Organization for Research and Treatment of Cancer (RTOG/EORTC) system [33]. Grade 1 complication was defined as slight dryness not affecting quality of life. Grade 2 complication was defined as moderate dryness that required a water bottle. Grade 3 complication was defined as severe dryness that caused a profound change in the quality of life. Xerostomia was assessed independently by 2 physicians (PXB and LY). Differences were resolved by discussion with a third physician (ZXD).

### Endpoints

The endpoint was xerostomia 1 year after treatment completion. The patients were divided into grade 0-1 and grade 2-3 xerostomia groups.

### Statistical analysis

Significant differences of clinical variables and dosimetric parameters between grade 0-1 and grade 2-3 xerostomia groups 1 year post-treatment were assessed. Continuous characteristics of age and dosimetric parameters were compared using Student’s t-test or Mann-Whitney U test for variables with an abnormal distribution. Categorical characteristics of sex, T stage, N stage, AJCC stage, pathology, body mass index, and chemotherapy were compared using the Chi-square test or Fisher’s exact test. The area under the curve (AUC) of the receiver operating characteristic (ROC) curve was used to assess the predicted probability. The predictive factors for xerostomia were analysed using logistic regression analysis. Combined predictor (mx) was calculated using the results of the multivariate analysis of logistic regression analysis. Statistical analyses were performed using SPSS Statistics Version 23.0 software (IBM Co., Armonk, NY, USA).

A nomogram was constructed based on the predictors combined with the clinical variables using the rms package in R version 3.5.3 (http://www.r-project.org/). A final model selection was performed by a backward stepdown selection process with the Akaike information criterion [34]. The performance of the nomogram was measured by a calibration plot. All P values were two sided. A P value < 0.05 was considered statistically significant.
